# A clinicopathological analysis of adrenal tumors in patients with history of extra-adrenal cancers

**DOI:** 10.1186/s12885-019-6061-y

**Published:** 2019-08-27

**Authors:** Lei Tan, Yunlin Ye, Kanghua Xiao, Xuelian Xu, Haitao Liang, Fufu Zheng, Zike Qin

**Affiliations:** 1 Department of Urology, State Key Laboratory of Oncology in South China, Collaborative Innovation Center for Cancer Medicine, Sun Yat-sen University Cancer Center, Guangzhou, 510060 Guangdong China; 2 Department of Urology, The First Affiliated Hospital of Sun Yat-Sen University, Guangzhou, 510080 Guangdong China

**Keywords:** Adrenal tumor, Extra-adrenal cancer, Predictor, Differentiation

## Abstract

**Background:**

Adrenal tumors in patients with previous/synchronous extra-adrenal malignancy are diverse and are a dilemma in clinical practice. This study investigated the differentiation of adrenal malignant and benign tumors in these patients.

**Methods:**

Data from patients with a pathological diagnosis of adrenal tumors were retrospectively retrieved from April 1991 to November 2015. Patients without extra-adrenal malignancy were excluded. Clinical and imaging characteristics, including sex, age, tumor size, tumor location, isolated lesion, time interval between the diagnosis of the two tumors and retrieved imaging diagnosis, were collected and analyzed. The selected patients were divided into 2 groups: those with primary or secondary malignancies (PSM) and those with primary benign tumors (PB). Chi-squared tests were used to evaluate differences between the two groups. Logistic regression was performed to explore potential risk factors related to the differentiation of PSM and PB, and a receiver operating characteristic (ROC) curve was used to evaluate their diagnostic values.

**Results:**

Ninety-one patients were selected; 54 were male, and the median age was 56 years old. Between the groups of PSM and PB, sex (*p* = 0.004), age (*p* = 0.029), tumor size (*p* < 0.001), isolated lesion (*p* < 0.001) and imaging diagnosis (*p* < 0.001) were significantly different, while tumor size (*p* = 0.001), sex (*p* = 0.047) and imaging diagnosis (*p* = 0.002) were independent predictors of PSM. With ROC curve analysis, risk factors ≥2 was the optimal cutoff to differentiate these adrenal tumors, and their sensitivity and specificity were 73 and 77%, respectively. With a median follow-up of 32 months, only 4 of 32 patients with PB died from cancer, and 24 of 47 patients with PSM died from cancer, although aggressive treatment was performed.

**Conclusions:**

Tumor size, sex and imaging diagnosis were independent predictors of adrenal primary or secondary malignancies. These predictors might be helpful for differentiation of adrenal tumors in patients with previous/synchronous extra-adrenal cancers.

## Background

The adrenal gland is a common site for metastasis of some malignancies because of its rich sinusoidal blood supply, as reported in autopsy reports [1, 2]. Fortunately, the rapid development and widespread application of modern imaging technologies has led to an increase in the diagnosed number of clinically silent adrenal masses [3–6]. The prevalence of primary adrenal tumors in patients with a history of extra-adrenal malignancy ranges from 27 to 50% [7]. It is still crucial to distinguish benign from malignant lesions and metastases in these suspicious masses. At the moment when the incidental adrenal mass is found with a history of any extra-adrenal malignant tumors, distinguishing the primary tumor from metastasis influences the management of these patients [8]. However, adrenal masses identified in a patient with previous/synchronous extra-adrenal malignancies are frequently deemed to be metastatic lesions [7]. However, if the mass is not a metastasis, the clinical decision is extremely different, so proper preoperative diagnostic studies are necessary. Additionally, the guidelines of adrenal incidentaloma in 2016 recommend evaluating an adrenal tumor as benign or malignant at initial management, and then sequential treatment is based on this decision [9]. Therefore, an incorrect diagnosis could result in inappropriate treatment of adrenal tumors: over treatment or overlooked. This study investigated the differentiation of adrenal malignant and benign tumors in patients with a history of extra-adrenal malignant tumors.

## Methods

From April 1991 to November 2015, patients with adrenal tumors and a history of extra-adrenal malignancy were retrieved from Sun Yat-sen University Cancer Center and The First Affiliated Hospital of Sun Yat-Sen University. In total, 183 patients with adrenal tumors had previous/synchronous extra-adrenal malignancies; then, those patients without pathological results were excluded, and ninety-one patients met our criteria. The clinical data, including sex, age, hormone evaluation, pathological diagnosis, interval time between diagnosis of the two tumors, tumor size and imaging characteristics, were retrospectively collected. From the pathological results, these adrenal tumors were categorized into 2 groups: primary or secondary malignancies (PSM) and primary benign tumors (PB). Based on radiological reports, tumors with a tumor density > 10 HU or enhancement of density > 20 HU on CT scan, a maximum standardized uptake value (SUVmax) > 4.0 on 18F-FDG PET/CT, nonuniform enhancement and irregular margins were considered as suspected adrenal malignancies.

For patients with isolated adrenal lesions, clinical and imaging characteristics were analyzed, and risk factors for predicting adrenal malignancy were investigated.

Based on IBM-SPSS Statistics®, version 20 (IBM-Corp., Armonk, NY), the Chi-square test was used to analyze the relationship between pathological diagnosis and clinical/radiological features. Univariate and multivariate logistic regression analyses were performed to explore risk factors for PSM, and a receiver operating characteristic (ROC) curve was used to evaluate their diagnostic values. All tests were two-tailed, and *p* < 0.05 was considered to be statistically significant.

## Results

Fifty-two patients with PSM and 39 with PB were investigated in this study. The median age was 56 years old (9 to 80 years), and 54 patients were male. Tumor size ranged from 0.5 to 12.0 cm (median, 2.7 cm) (Table 1). In 3 patients with primary malignancy, 2 had adrenal cortical carcinoma and 1 had neuroendocrine carcinoma, with a size range from 6.1 cm to 12.0 cm. In 39 patients with PB, 24 had cortical adenoma, 10 had pheochromocytoma, 3 had nodular hyperplasia, 1 had ganglioneuroma and 1 had no tumor.

**Table 1:** Clinical and imaging characteristics of patients with PSM and PB

Clinical and imaging feature	Total (%)	PB (*n* = 39)	PSM (*n* = 52)	*P* value
Age (years), median (range)	56(9–80)	52 (23–80)	58(9–75)	0.070
≤ 55	44 (48.4)	24(54.5)	20(45.5)	0.029
> 55	47 (51.6)	15(31.9)	32(68.1)	
Gender				
Female	38(41.8)	23 (60.5)	15 (39.5)	0.004
Male	53 (58.2)	16 (30.2)	37 (69.8)	
Location				
Right	37(40.7)	20(54.1)	17(45.9)	0.201
Left	42(46.2)	15(35.7)	27(64.3)	
Bilateral	12(13.2)	4(33.3)	8(66.7)	
Size (cm), median (range)	2.7(0.5–12.0)	2.3(0.9–5.7)	3.4(0.5–12.0)	0.001
<3.2	56(61.5)	32 (57.1)	24 (42.9)	< 0.001
≥ 3.2	35(38.5)	7 (20.0)	28 (80.0)	
Isolated lesion				
No	22 (24.2)	1 (4.5)	21 (95.5)	< 0.001
Yes	69 (75.8)	38 (55.1)	31 (44.9)	
Imaging diagnosis				
Malignant	61 (67.0)	17 (27.9)	44 (72.1)	< 0.001
Benign	30 (33.0)	22 (73.3)	8 (26.7)	
Interval time (month), median (range)	15.5 (0–146)	9(0–146)	18.5(0–91)	0.620

*PSM* primary or secondary malignancies found in patient with history of extra-adrenal malignancy

*PB* primary benign tumors found in patient with history of extra-adrenal malignancy

Hormonal evaluation was performed in 56 patients, 6 of whom were diagnosed with pheochromocytoma, 7 with hypercortisolism, 1 with subclinical Cushing syndrome, 1 with aldosteronism, 2 with adrenocortical hyposecretion, and 39 with nonfunctional tumors.

The most common extra-adrenal cancers were lung carcinoma, followed by gastrointestinal (15 colorectal cancer and 2 gastric cancer), hepatic and urological cancers (Table 2). In the others group, esophageal cancer and ovarian cancer were 2, cervical cancer, nasopharyngeal carcinoma, neuroendocrine carcinoma, pancreatic carcinoma, submandibular neuroblastoma, testicular stroma and thymic carcinoma were 1. Adrenal metastasis was most common in patients with primary lung and liver cancer, and no metastasis was detected in patients with thyroid cancer.

**Table 2:** Clinical characters of patients stratified by extra-adrenal malignancy

Cancer diagnosis	No.	Age (year), Mean (range)	GenderMale: Female	Mass size (cm)		Metastasis: Primary malignancy: PB	Surgical approaches Open: Laparoscopy
				Median (range)	< 3.2:≥3.2		
Lung cancer	20	58(36–70)	14: 6	2.7(1.5–9.5)	13: 7	17: 0: 3	12: 7
Gastrointestinal cancer	17	59(43–75)	9: 8	2.3(0.5–6.5)	14: 3	7: 0: 10	7: 8
Liver cancer	15	56(34–80)	11: 4	2.8(1.4–7.7)	8: 7	11: 1: 3	7: 7
Urinary cancer	13	53(34–75)	9: 4	3.0(0.5–10.5)	8: 5	6: 0: 7	2: 10
Thyroid cancer	9	36(23–51)	4: 5	2.8(0.9–5.7)	5: 4	0: 0: 9	6: 3
Breast cancer	6	53(42–65)	0: 6	2.8(2.0–12.0)	4: 2	1: 1: 4	3: 2
The others	11	48(9–66)	7: 4	4.2(1.0–8.1)	4: 7	7: 1: 3	4: 7
Total	91	53(9–80)	54: 37	2.7(0.5–12.0)	56: 35	49: 3: 39	41: 44*

* Six patients underwent biopsy only

*PB* primary benign tumors found in patient with history of extra-adrenal malignancy

According to comparative analysis, tumor size, sex and age were different between PSM and PB. The overall median interval time between extra-adrenal cancer and adrenal lesion was 15.5 months (0 to 146 months), and the median time interval of PSM was almost twice that of PB (18.5 vs 9 months), although this difference was not significant (*p* = 0.620). An ROC curve was performed to find the optimal cut-off value of tumor size, which was 3.2 cm. For tumor size ≥3.2 cm or < 3.2 cm, the proportions of malignant tumors were 80 and 43%, respectively.

Most PB were isolated lesions, and extra-adrenal malignancies with isolated adrenal metastasis were associated with better survival when the adrenal lesion was dissected. So how to differentiate patients with adrenal PSM or not was of clinical significance to isolated adrenal lesions. The association of PSM and PB with the clinical and imaging characteristics was analyzed for those with solitary adrenal masses (Table 3). There were 38 patients with PB and 31 with PSM. With a median follow-up of 32 months, only 4 patients with PB died from cancer,including 1 breast cancer, 1 ovarian cancer, 1 kidney cancer and 1 bladder cancer. And 24 with PSM died from cancer, although aggressive treatment was performed, including 10 lung cancer, 5 colorectal cancer, 4 liver cancer, 3 urinary cancer, 1 breast cancer and 1 esophageal cancer.

**Table 3:** Clinical and imaging characteristics of patients with solitary adrenal mass

Clinical and imaging feature	Total (%)	PB (*n* = 38, 55.1%)	PSM (*n* = 31, 44.9%)	*P* value
Age (years)				0.071
≤ 55	35 (50.7)	23(65.7)	12(34.3)	
> 55	34 (49.3)	15(44.1)	19(55.9)	
Gender				0.019
Male	36 (52.2)	15 (41.7)	21 (58.3)	
Female	33 (47.8)	23 (69.7)	10 (30.3)	
Size				< 0.001
< 3.2 cm	43(62.3)	31 (72.1)	12 (27.9)	
≥ 3.2 cm	26(37.7)	7 (26.9)	19 (73.1)	
Imaging diagnosis				< 0.001
Malignant	42 (60.9)	16 (38.1)	26 (61.9)	
Benign	27 (39.1)	22 (81.5)	5 (18.5)	
The number of risk factors				
< 1	10 (14.5)	10 (100)	0 (0.0)	0.002
≥ 1	59 (85.5)	28 (47.5)	31 (52.5)	
< 2	36 (52.2)	29 (80.6)	7 (19.4)	< 0.001
≥ 2	33 (47.8)	9 (27.3)	24 (72.7)	
< 3	57 (82.4)	37 (64.9)	20 (35.1)	< 0.001
≥ 3	12 (17.6)	1 (8.3)	11 (91.7)	

*PSM* primary or secondary malignancies found in patient with history of extra-adrenal malignancy

*PB* primary benign tumors found in patient with history of extra-adrenal malignancy

**Fig. 1:**
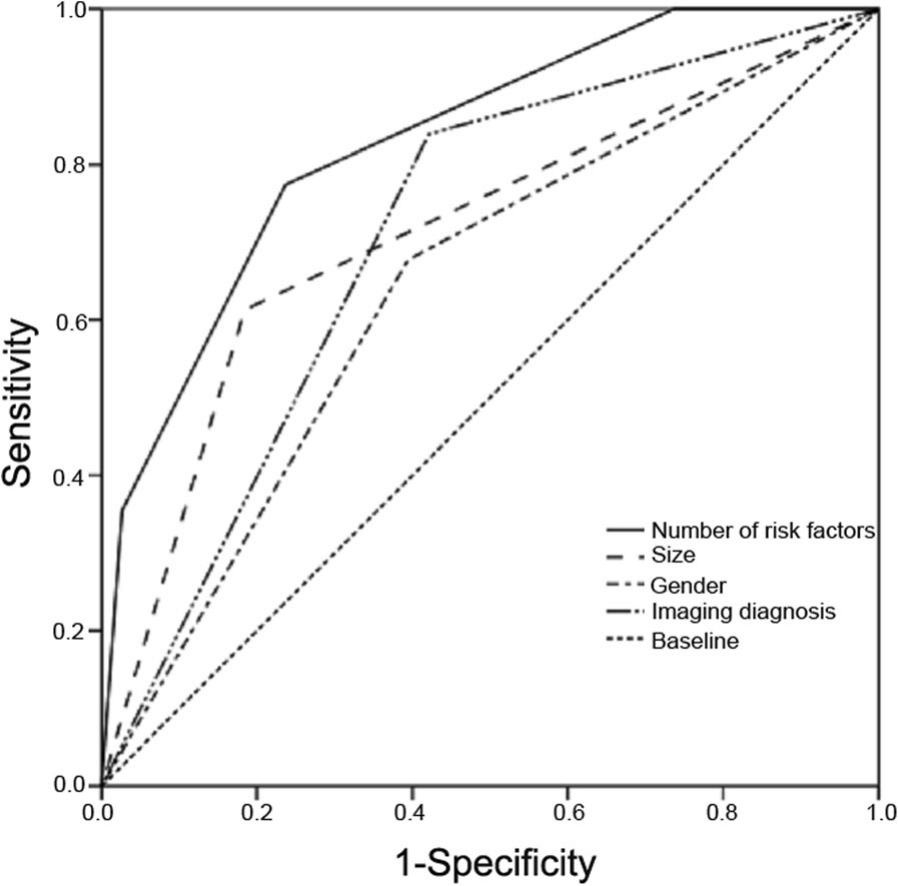
ROC curve of the selected risk factors responsible for the solitary PSM adrenal mass. PSM: primary or secondary malignancies found in patient with history of extra-adrenal malignancy

Multivariate logistic regression analysis showed that tumor size, sex and imaging diagnosis were independently associated with PSM in patients with solitary adrenal tumors, regardless of age and location (*p* = 0.074 and 0.908, respectively) (Table 4). Consequently, we considered a better approach based on the number of risk factors, which included tumor size, sex and imaging diagnosis. To compare the predictive value of the new approach with the single risk factor, we performed an ROC curve analysis (Fig. 1). The area under curve (AUC) of the new approach was 0.830, which was higher than the AUC of the other approaches (95% CI: 0.735–0.925; *P* < 0.001); the optimal cut-off value was 2 (sensitivity: 77.4%; specificity: 76.3%). Of the 33 cases with risk factors ≥2, 24 were PSM; when the cut-off was 1, the sensitivity and specificity were 100 and 26%, respectively.

**Table 4:** Univariate and multivariate logistics analyses of the clinical and imaging features for PSM in patients with solitary adrenal tumors

Diagnostic parameter	Univariate analysis *P* value	Multivariate analysis	
		Hazard(95% CI)	*P* value
Age	0.074		
< 55 yr			
≥ 55 yr			
Gender	0.021	3.552(1.015–12.429)	0.047
Male			
Female			
Location	0.908		
Unilateral			
Bilateral			
Size	< 0.001	9.835(2.521–38.363)	0.001
< 3.2 cm			
≥ 3.2 cm			
Imaging diagnosis	0.001	7.378(1.923–28.312)	0.004
Malignant			
Benign			

*PSM* primary or secondary malignancies found in patient with history of extra-adrenal malignancy

## Discussions

Adrenal glands are the fourth most common site of metastases in malignant diseases [10]. Abrams et al. found that 27% of cases involved the adrenal glands in 1000 consecutive autopsies of patients with carcinoma [11]. Furthermore, it was reported that approximately 50–75% of clinically imperceptible adrenal masses of cancer patients were metastases [7, 12–14]. The general assumption that an adrenal mass in a patient with concurrent or prior extra-adrenal cancer is a metastasis rather than a primary tumor may result in unnecessary and inappropriate cancer-directed management in clinical practice [7]. Therefore, in 2016, the guidelines for adrenal incidentaloma recommended that adrenal masses should be diagnosed as benign or malignant at the beginning of management of patients with extra-adrenal malignancy [9]. In addition, differentiation between PSM and PB is a critical problem in the clinical management of these adrenal tumors, especially for patients with isolated adrenal lesions, because adrenalectomy can offer increased survival outcomes when the lesion is isolated in the gland [15].

In our study, 57% of patients had PSM, which is in accordance with previous reports. Most of these patients had metastases, and only 3 had primary malignancies. There were still 39 patients with benign masses who were at potential risk of over-treatment, although some of the masses consisted of active hormones. Similar to the results of our previous report, most solitary adrenal lesions were benign tumors, and functional tumors were not rare, including pheochromocytoma [16]. In this study, more than 30% patients didn’t undergo hormonal evaluation, and pheochromocytoma and hypercortisolism presented about 25% in these patients with hormonal investigation, so misdiagnosis might lead to severe surgical complications. Thus, hormonal evaluation should be performed regularly as recommended by recent guidelines. For these patients with solitary adrenal tumors, diagnostic analysis demonstrated that sex, tumor size and imaging diagnosis were independent predictors of PSM. Compared to the index of any single risk factor, the comprehensive risk factor index (> 1 risk factors) had better diagnostic value for differentiating PSM from PB, and its sensitivity and specificity were 77 and 76%, respectively. Importantly, with a median follow-up of 32 months, over 50% of patients with PSM died of cancer, which was much higher than those with PB, although aggressive treatment was performed. For patients with solitary adrenal metastasis, resection could improve their oncological outcomes. Therefore, it is critical to differentiate malignant tumors from benign tumors for adrenal tumors with extra-adrenal cancers.

In clinical practice, the tumor size and imaging diagnosis play important roles in the differentiation between benign and malignant adrenal tumors. For adrenal tumors with extra-adrenal malignancy, these two factors are still helpful. In this study, the sensitivity of imaging diagnosis was about 85%, which was little lower than that of previous report. Most of adrenal malignancies (49/52) were metastasis in this study, and this status might affect the diagnostic value of imaging. What’s more, a threshold of 4 cm is often used as an indication of adrenalectomy, and our study showed that a threshold of 3.2 cm was proven to have the highest diagnostic value in this cohort. Compared to male patients, female patients were more likely to have benign adrenal tumors. Overall, these three independent factors were related to PSM in patients with previous/synchronous extra-adrenal malignancies. In fact, the cut-off of 2 risk factors had the best diagnostic efficacy, but the sensitivity was unsatisfactory, with a false-negative > 20%. It is possible that a cut-off of 1 risk factor with a sensitivity of 100% would be better in clinical practice.

Jeffrey T. Lenert et al. found that renal cell carcinoma was the most common origin of adrenal metastasis in patients with a history of extra-adrenal malignancy (30.8%) from 1971 to 2000 [7]. Lung cancers predominantly outnumbered renal cancers in our study, and the proportions of lung and renal cancer were 22 and 11%, respectively. Selection bias might have also played a role in this study, and patients with multi-organ metastasis, which is often determined by easy-access to organs other than the adrenal gland, were not included in this study.

In recent years, several studies have focused on the diagnosis of adrenal tumors. Our previous study revealed that malignant and functional tumors are not rare in patients with adrenal incidentaloma and that a history of other cancers is not associated with adrenal malignancy [16]. Byeon et al. and colleagues demonstrated that high pre-contrast HU, male sex, and metachronous adrenal mass were associated with malignant adrenal lesions, which was similar to the results of our study [17]. However, this finding is far from perfect, and fine needle aspiration could be a reliable examination method for selected patients, although there is a potential risk of hypertensive crisis [10]. Our study determined that a comprehensive analysis of these risk factors could improve the evaluation of adrenal tumors in patients with other cancers at initial management.

Because of the retrospective nature of this study, which represented more than 12 years of clinical experience, some patients didn’t have hormonal evaluation in this study, and imaging evaluations were not necessarily standardized, so we were not able to examine the explicit attenuation values. The small population and selection biases in these two medical centers were also limitations. However, these two affiliated institutes are the largest medical centers in South China, which may represent a good profile of adrenal tumors in patients with other cancers. Further research with prospective and multicenter studies would be useful to guide the understanding of patients with adrenal masses and a history of known extra-adrenal malignancies.

## Conclusions

Tumor size > 3.2 cm, male sex and malignant imaging diagnosis were independent predictive factors for solitary PSM, and a new index with a number of these risk factors improved differential diagnosis. Further research with prospective studies would be useful to guide the management of patients with incidentally discovered adrenal masses and a history of extra-adrenal cancer.

## Data Availability

The datasets generated and/or analysed during the current study are available in the [Research Data Deposit public platform] repository, [http://www.researchdata.org.cn]. The approval RDD number is RDDA2019001107.

## Abbreviations

PB: primary benign tumors found in patient with history of extra-adrenal malignancy
PSM: primary or secondary malignancies found in patient with history of extra-adrenal malignancy
ROC curve: receiver operating characteristic curve
